# Development and specification of cerebellar stem and progenitor cells in zebrafish: from embryo to adult

**DOI:** 10.1186/1749-8104-8-9

**Published:** 2013-05-04

**Authors:** Jan Kaslin, Volker Kroehne, Francesca Benato, Francesco Argenton, Michael Brand

**Affiliations:** 1 Biotechnology Center and Center for Regenerative Therapies Dresden, Dresden University of Technology, Fetscherstr. 105, Dresden, 01307, Germany; 2 Australian Regenerative Medicine Institute, Monash University, Wellington Road, Melbourne, 3800, Australia; 3 Dipatimento di Biologia dell’Università degli Studi di Padova, Via U. Bassi 58b, Padova, 35131, Italy

**Keywords:** Cerebellum, Glia, Granule cell, Neural stem cell, Neurogenesis, Niche, Teleost, Regeneration, Upper rhombic lip, Ventricular zone

## Abstract

**Background:**

Teleost fish display widespread post-embryonic neurogenesis originating from many different proliferative niches that are distributed along the brain axis. During the development of the central nervous system (CNS) different cell types are produced in a strict temporal order from increasingly committed progenitors. However, it is not known whether diverse neural stem and progenitor cell types with restricted potential or stem cells with broad potential are maintained in the teleost fish brain.

**Results:**

To study the diversity and output of neural stem and progenitor cell populations in the zebrafish brain the cerebellum was used as a model brain region, because of its well-known architecture and development. Transgenic zebrafish lines, *in vivo* imaging and molecular markers were used to follow and quantify how the proliferative activity and output of cerebellar progenitor populations progress. This analysis revealed that the proliferative activity and progenitor marker expression declines in juvenile zebrafish before they reach sexual maturity. Furthermore, this correlated with the diminished repertoire of cell types produced in the adult. The stem and progenitor cells derived from the upper rhombic lip were maintained into adulthood and they actively produced granule cells. Ventricular zone derived progenitor cells were largely quiescent in the adult cerebellum and produced a very limited number of glia and inhibitory inter-neurons. No Purkinje or Eurydendroid cells were produced in fish older than 3 months. This suggests that cerebellar cell types are produced in a strict temporal order from distinct pools of increasingly committed stem and progenitor cells.

**Conclusions:**

Our results in the zebrafish cerebellum show that neural stem and progenitor cell types are specified and they produce distinct cell lineages and sub-types of brain cells. We propose that only specific subtypes of brain cells are continuously produced throughout life in the teleost fish brain. This implies that the post-embryonic neurogenesis in fish is linked to the production of particular neurons involved in specific brain functions, rather than to general, indeterminate growth of the CNS and all of its cell types.

##  Background

During the development of the vertebrate central nervous system (CNS) different cell types are produced in a strict temporal order from increasingly committed progenitors. Diverse cells with stem and progenitor potential have been observed *in vivo* and *in vitro* in the post-embryonic brain [1-4]. The heterogeneity and nature of neural stem cells *in vivo* is poorly understood. For example, it is currently debated whether neural stem cells in the rodent brain are disposable or maintained indefinitely [5]. In addition, it is unclear whether the stem and progenitor cells that persist into adulthood retain the capacity to produce all the cell types in the tissue or if only specific lineages of cells are produced.

**Figure 1:**
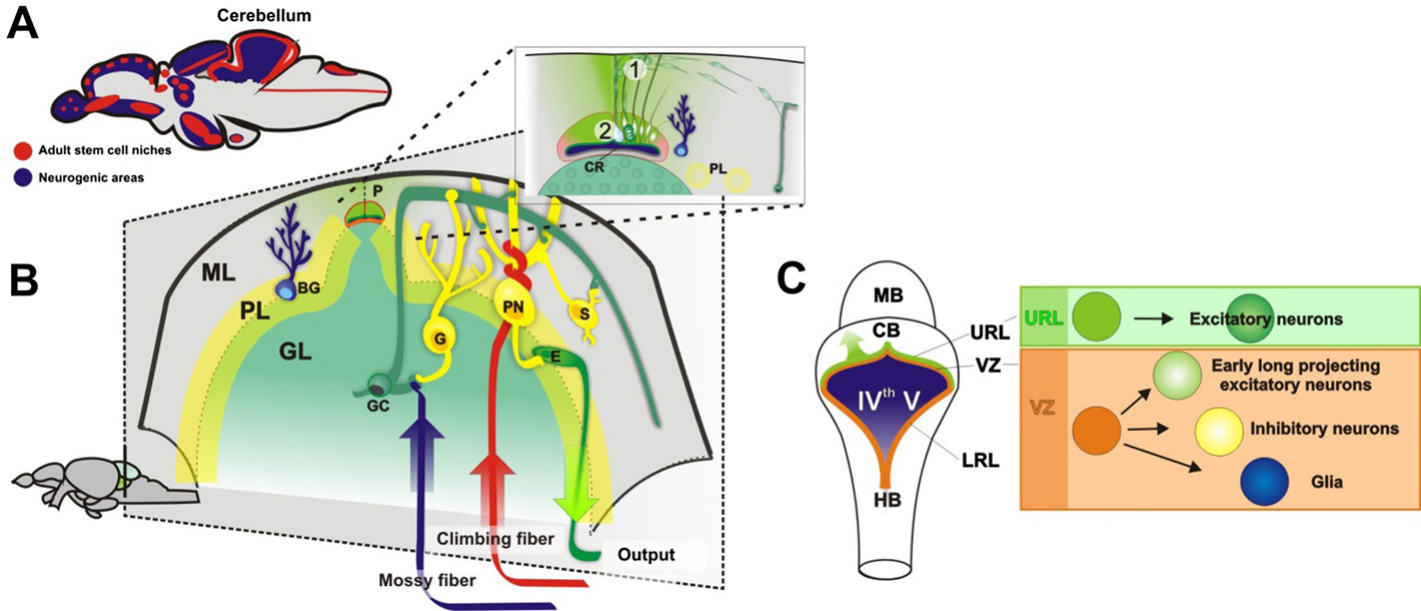
**Overview of the cerebellar architecture in zebrafish.** (**A**) In the adult zebrafish brain neural stem cells are abundant and distributed in distinct topological clusters along the whole rostro-caudal brain axis; (**B**) A schematic coronal section showing the anatomy of the zebrafish cerebellum. The cerebellum has a simple laminar three layered architecture consisting of a molecular layer (ML), Purkinje cell layer (PL) and a granule cell layer (GL). The granule layer consists of excitatory granule cells and inhibitory Golgi neurons. The Purkinje cell layer contains Purkinje neurons (PN), Bergmann glia (G), and excitatory eurydendroid cells (E). The ML mainly consists of nerve fibers and scattered inhibitory stellate cells. *Boxed region*. Neural progenitors are maintained in the dorsomedial part of the cerebellum around a remnant of the IV^th^ ventricle (the cerebellar recessus, CR). The progenitors give rise to granule neurons in a distinct outside-in fashion. (**1**) Polarized neuroepithelial-like progenitors (green) are restricted to the midline of the dorsal cerebellum. The progenitors give rise to rapidly migrating granule precursors (dark green) that initially migrate dorsolaterally. After reaching the meninge the granule precursors migrate in ventrolateral direction to the GL. (**2**) Radial glia (light blue) are found close to the midline and they are used as scaffolds during the initial dorsal migration of granule precursors. **C**) Schematic view of the cerebellar progenitor domains during development. The cerebellar cell types are generated from two germinal zones: the ventricular zone (VZ, orange) and the upper rhombic lip (URL, green). The excitatory neurons are generated from the URL, while inhibitory neurons and glia are generated from the VZ. The cerebellar cell types are produced in a strict temporal order from increasingly committed progenitors. Purkinje neurons and deep cerebellar nuclei neurons are produced early during development, while inhibitory interneurons, granule cells and glia are produced late.

Teleost fish display widespread post-embryonic neurogenesis and undetermined brain growth throughout life [6-14]. The widespread neurogenesis originates from many different proliferative niches that are distributed along the brain axis (Figure 1A). This makes teleost fish an exciting model to study neuronal stem and progenitor cell diversity. Different neural progenitor types based on cellular morphology, molecular marker characteristics and fibroblast growth factor (Fgf) signaling requirements are found in the zebrafish brain, suggesting that different stem and progenitor cell populations are retained into adulthood [15-21]. However, it is currently not known whether diverse neural stem and progenitor cell types with restricted potential or stem cells with broad potential are maintained in the teleost fish brain.

The zebrafish cerebellum has a well-known architecture, cell types and development (Figure 1A), making it an excellent model region to study the diversity and output of neural stem and progenitor cell populations. [19,22-25]. The cerebellum contains relatively few cell types with distinct morphological, molecular and physiological characteristics (Figure 1B). The cerebellar neurons can be divided into two major categories based on their function as inhibitory or excitatory. The inhibitory neurons use gamma-butyric acid (GABA) or glycin as their main neurotransmitters, while the excitatory neurons use glutamate. During mammalian development, different cerebellar cell types are produced in a strict temporal order from increasingly committed progenitors (Figure 1C) [26]. Purkinje neurons and deep cerebellar nuclei neurons are produced early during development, while inhibitory interneurons, granule cells and glia are produced late [27]. In all vertebrates studied, including zebrafish, excitatory neurons are generated by progenitors of the upper rhombic lip (URL), while inhibitory neurons and glia are generated from progenitors in the ventricular zone (VZ, Figure 1C) [25,27]. The principal germinal zones of the zebrafish cerebellum have recently been thoroughly studied with molecular marker analysis and transgenic reporter lines labeling different progenitor populations [19,22,23,25,28-31,42]. We have previously shown that neuroepithelial-like progenitors in the zebrafish cerebellum are maintained into adulthood in a distinct niche [19]. However, it is not known if diverse cerebellar progenitor cell types remain active in the adult cerebellum and how the cerebellar progenitor niche progresses from embryo to adult.

By taking advantage of transgenic zebrafish lines, *in vivo* imaging and molecular markers to study the temporal dynamics of cerebellar progenitor cell populations and their output in the cerebellum of juvenile and adult zebrafish, we show that distinct stem and progenitor cell populations arise early during development. The proliferative activity and progenitor marker expression declines in juvenile zebrafish before the fish reach sexual maturity and this correlates with a diminished repertoire of cell types produced in the adult. The stem and progenitor cells derived from the URL are maintained into adulthood and they actively produce granule cells. VZ derived progenitor cells are largely quiescent in the adult cerebellum and produce a very limited number of glia and inhibitory inter-neurons. No Purkinje or Eurydendroid cells are produced in fish older than 3 months. This suggests that cerebellar cell types are generated in a strict temporal order from distinct pools of increasingly committed stem and progenitor cells.

## Results

### Nestin:egfp+ and Ptf1a:DsRed+ cell populations form complementary abutting cell populations in the cerebellar primordium

**Figure 2:**
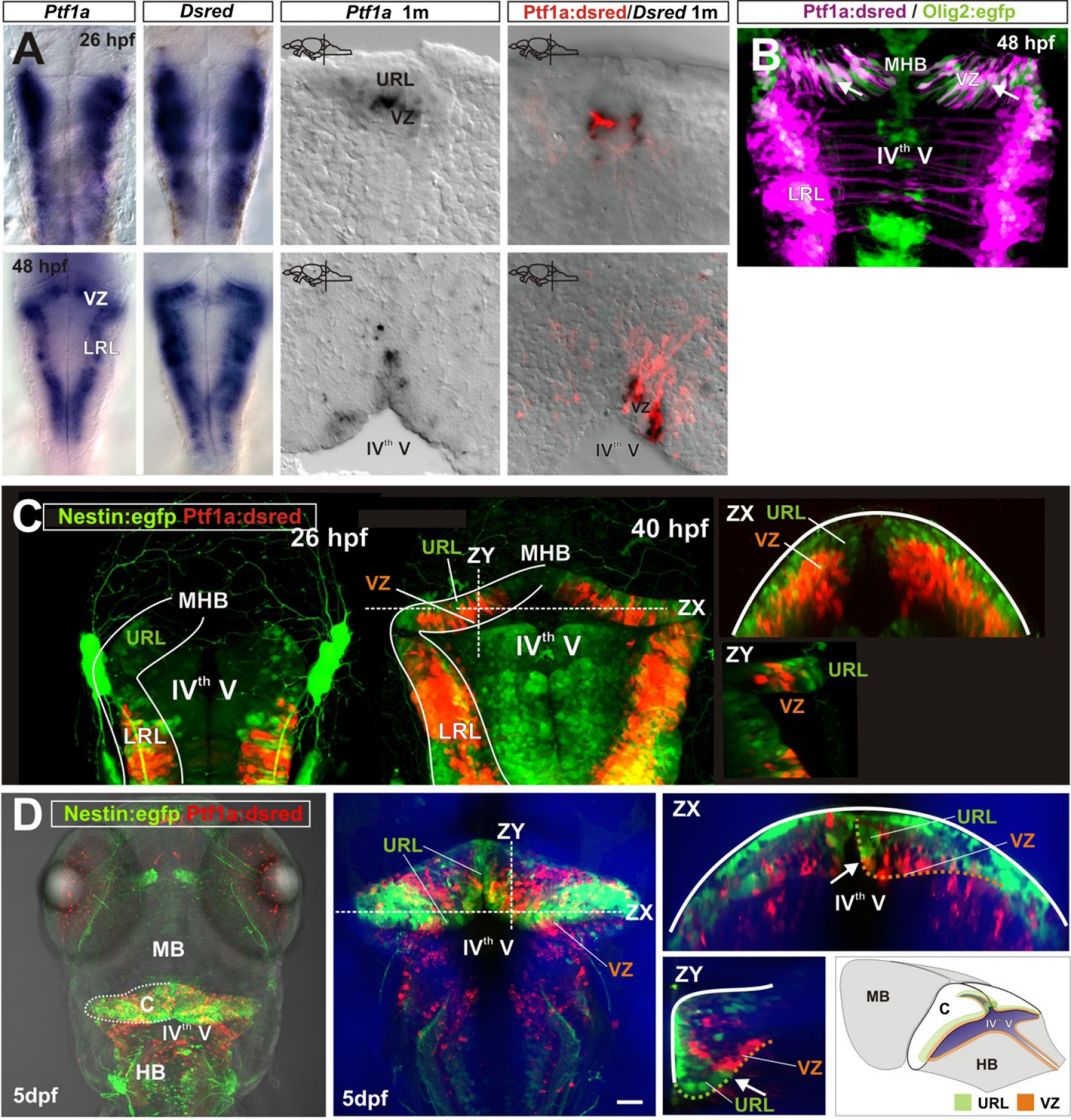
**Distinct cerebellar progenitor populations are established early in the embryo.** (**A**) Expression of *ptf1a*, *DsRed* (mRNA) and DsRed (protein) in developing and juvenile *Ptf1a:DsRed* transgenic fish. *Ptf1a, DsRed* and DsRed show similar expression patterns; (**B**) *In vivo* expression of DsRed and Egfp in the embryonic and juvenile *Ptf1a:DsRed* and *Olig2:egfp* transgenic fish. Overlapping *Egfp* and *DsRed* expression is seen in the VZ of the cerebellar primordium; (**C**) *Nestin:egfp*+ (green) and *Ptf1a:DsRed*+ (red) progenitors in the cerebellar primordium. The *Nestin:egfp* labels cells in the URL, while *Ptf1a:DsRed* line labels cells in the VZ. Two days post-fertilization *Nestin:egfp*+ and *Ptf1a:DsRed*+ cells form distinct populations in the URL and VZ of the cerebellar primordium; (**D**) A dorsal overview of *Nestin:egfp*+ (green) and *Ptf1a:DsRed*+ (red) progenitors in the hindbrain of a 5-day-old larval zebrafish. Two distinct progenitor domains are visible in the cerebellum (the junction is indicated with an arrow). *Ptf1a:DsRed*+ cells localize along the ventricular zone of the IV^th^ ventricle (the VZ domain is labeled with a hatched orange line), while *Nestin:egfp*+ cells localize in the URL (hatched green line). MHB: Mid-hindbrain boundary; LRL: Lower rhombic lip.

We performed a time course series from larval to adult stages and quantified the proliferative activity of the different cerebellar stem and progenitor cell types using transgenic reporter lines that label cerebellar progenitors in the URL and VZ. The transcription factor Ptf1a is expressed in VZ progenitors during cerebellar development in vertebrates and is necessary for neurons to adopt an inhibitory fate, thus, Ptf1a expression can be used as a readout for VZ progenitor activity [32,33]. We created a transgenic line in which a 5.5 kb 5’ fragment of the *Ptf1a* promoter drives the expression of *DsRed*. The expression pattern of *DsRed* in the *Tg(−5.5Ptf1a:DsRed)* transgenic line (referred from here on as *Ptf1a:DsRed*) was similar to the endogenous expression of *Ptf1a* in the VZ of the hindbrain of zebrafish, in accordance with previous reports (Figure 2A, [25,29]). The *Tg(Olig2:egfp)* transgenic line (referred from here on as *Olig2:egfp*) was used to label VZ progenitors that give rise to eurydendroid cells and oligodendrocytes [23,25,34]. In agreement with previous studies, the population of *Ptf1a:DsRed* expressing cells was found to overlap with *Olig2:egfp* expressing cells in the VZ of the zebrafish embryo (Figure 2B, [25]). The *Tg(Nestin:egfp)* transgenic line (referred from here on as *Nestin:egfp*), which labels neuroepithelial cells in the neural tube during embryonic stages and later specific progenitors in the brain [19], was used to follow neural progenitors in the URL. During embryonic stages, *Nestin:egfp* expression was widespread in the neural tube, but later in the cerebellar primordium (>48 hpf) the expression was restricted to the progenitors of the URL: The *Nestin:egfp*+ cells were distinct from the *Ptf1a:DsRed*+ cells in the VZ (Figure 2C). In larval fish at five days post-fertilization, the *Nestin:egfp*+ and *Ptf1a:DsRed*+ cells formed two distinct and complementary abutting cell populations in the URL and VZ, respectively (Figure 2D).

### The proliferative activity of the URL is maintained into adulthood

**Figure 3:**
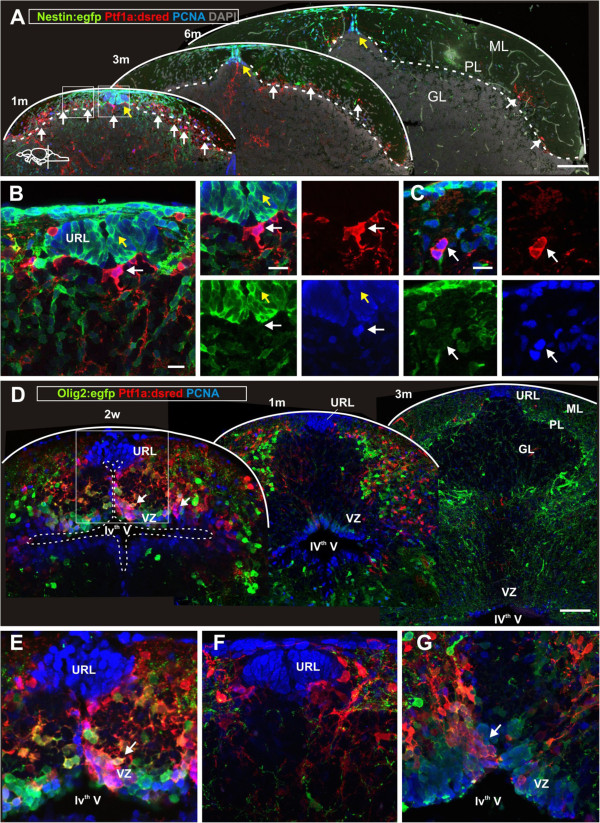
**Proliferative activity of cerebellar progenitors diminishes during juvenile stages.** (**A**) Confocal maximum projection of cerebellar cross sections at a similar level of juvenile, young and adult cerebellum. Proliferating cells are labeled with proliferating cell nuclear antigen (PCNA) in blue. *Nestin:egfp*+ cells are green, *Ptf1a:DsRed*+ cells are red and the general tissue morphology is depicted with DAPI staining in grey. Yellow arrows depict the distinct niche in the URL where proliferating *Nestin:egfp*+/PCNA+ progenitors are located. White arrows depict *Ptf1a:DsRed*+ cells that localize to the ventral part of the IV^th^ ventricle and to the cerebellar parenchyma. The majority of the parenchymal *Ptf1a:DsRed*+ cells are PCNA- while most of the *Ptf1a:DsRed*+ cells at the VZ close to the ventricle are PCNA+. (**B-C**) High magnification single confocal planes of the boxed areas in **A**. (**B**) A proliferating *Ptf1a:DsRed*+ /PCNA+ progenitor at the IV^th^ ventricle (white arrow) and many proliferating *Nestin:egfp*+/PCNA+ cells in the URL (yellow arrow); (**C**) A proliferating *Ptf1a:DsRed*+ cell in the cerebellar parenchyma (white arrow); (**D**) Confocal maximum projections of juvenile and adult cerebellar cross sections showing a decline in VZ progenitor activity. (**E**) High magnification of boxed area in **D**. Proliferating *Ptf1a:DsRed*+ (red) and *Olig2:egfp*+ (green) cells are found in VZ and the cerebellar parenchyma in a two-week-old juvenile fish. Note the absence of *Ptf1a:DsRed*+ and *Olig2:egfp*+ cells among the PCNA+ cells in the URL. The white arrow depicts the *Ptf1a:DsRed*+ and *Olig2:egfp*+cells laminating from the ventricular surface into the parenchyma; (**F**) *Ptf1a:DsRed*+ cells adjacent to proliferating PCNA+ cells in a one-month-old fish; (**G**) Proliferating *Ptf1a:DsRed*+ and *Olig2:egfp*+ in the VZ of the fourth ventricle (arrow) in a one-month-old fish. The white arrow shows the *Ptf1a:DsRed*+ and *Olig2:egfp*+ cells laminating from the ventricular surface into the parenchyma.

**Figure 4:**
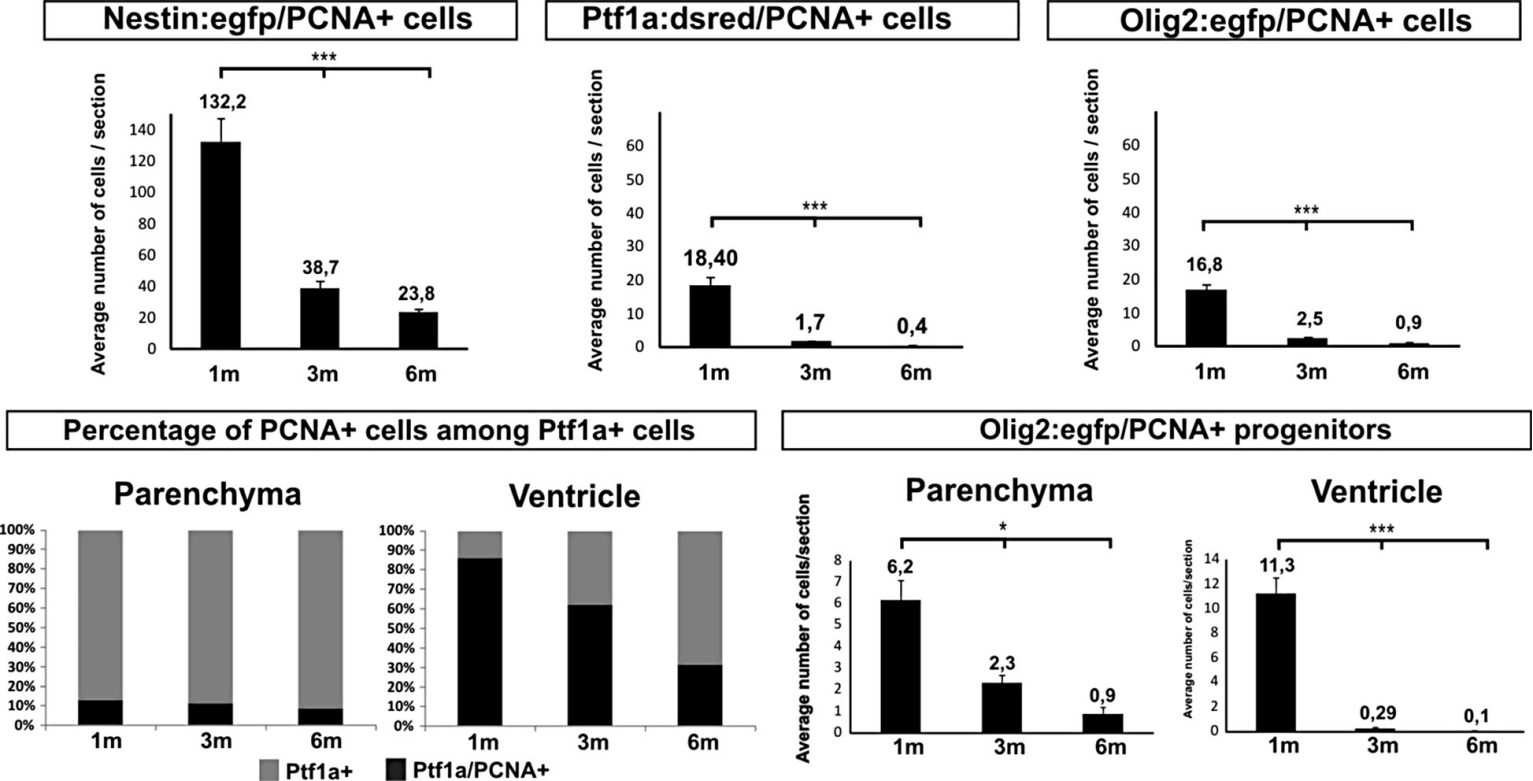
**Quantification of the proliferative activity of cerebellar progenitors.** The number of proliferating *Nestin:egfp*+ progenitors are significantly reduced during juvenile stages but notable progenitor activity is still detected in the adult and aging brain (*P* <0.001, n = 5). A significant loss of the proliferative activity of *Ptf1a:DsRed*+ and *Olig2:egfp*+ cells takes place during juvenile stage and the activity is almost exhausted in the adult (*Ptf1a:DsRed*: *P* <0.001, n = 7; *Olig2:egfp*: *P* <0.001, n = 5). The proliferating *Olig2:egfp* cells show a similar decline in the proliferative activity at the ventricle and in the brain parenchyma as the *Ptf1a:DsRed*+ cells.

To determine if progenitors in the URL and VZ maintain their proliferative activity beyond larval stages, we performed a quantitative co-localization study using the transgenic lines and the cell proliferation marker proliferating cell nuclear antigen (PCNA) in juvenile fish (1 month), young adults (3 months) and adult fish (6, 14 and 22 months). *Nestin:egfp*+ progenitors were maintained in a distinct niche in the dorsomedial cerebellum from larval stages to the adult (yellow arrow, Figure 3A). Co-localization of *Nestin:egfp+* cells and PCNA+ showed that the majority of the *Nestin:egfp*+ cells were proliferating (>95%, n = 5, Figure 3B). The number of PCNA+ and *Nestin:egfp*+ progenitors showed a significant decline in number in the transition from juvenile to adult fish (Figure 4). However, a notable proliferative activity of *Nestin:egfp*+ progenitors was still detected in the adult and aging cerebellum (*P* <0.001, n = 5, Figure 4).

### Loss of proliferative activity of VZ progenitors takes place from the juvenile to adult stages

In juvenile fish the *Ptf1a:DsRed*+ and *Olig2:egfp*+ progenitors continued to proliferate along the VZ of the IV^th^ ventricle (Figure 3D). The highest density of proliferating *Ptf1a:DsRed*+ and *Olig2:egfp*+ cells were found in the posterior part of the cerebellum, at the interface to the caudal lobe (Figure 3G). In two- and four-week-old juveniles, cohorts of *Ptf1a:DsRed*+ and *Olig2:egfp*+ cells appeared to delaminate from the ventricular surface of the VZ into the cerebellar parenchyma (Figure 3D,E). Proportionally, most of the proliferating PCNA+ and *Ptf1a:DsRed*+ or *Olig2:egfp*+ cells were found at the ventricle (Figure 4). The proliferative activity of *Ptf1a:DsRed*+ and *Olig2:egfp*+ progenitors diminished significantly with age, and proliferating *Ptf1a:DsRed*+ or *Olig2:egfp*+ cells were almost absent in the adult cerebellum (*P* <0.001, Figure 4). In particular, the proliferative activity of *Ptf1a:DsRed*+ and *Olig2:egfp*+ cells declined within the VZ. In juvenile and young fish, the majority of the *Ptf1a:DsRed*+ cells at the ventricle proliferated, in contrast to adult fish, where most of the *Ptf1a:DsRed*+ expressing cells were quiescent (Figure 4). The proliferative quiescence was accompanied by changes in morphology from a radial to a multipolar and flat epithelial morphology (see below). A small subpopulation of the *Ptf1a:DsRed*+ and *Olig2:egfp*+ cells continued to proliferate in the cerebellar parenchyma of juvenile and young fish (Figure 3C) but in the adult parenchymal cell proliferation was almost absent (Figure 3A).

### Nestin:egfp, Ptf1a:DsRed and Olig2:egfp expressing progenitors give rise to distinct lineages of cells in the cerebellum

**Figure 5:**
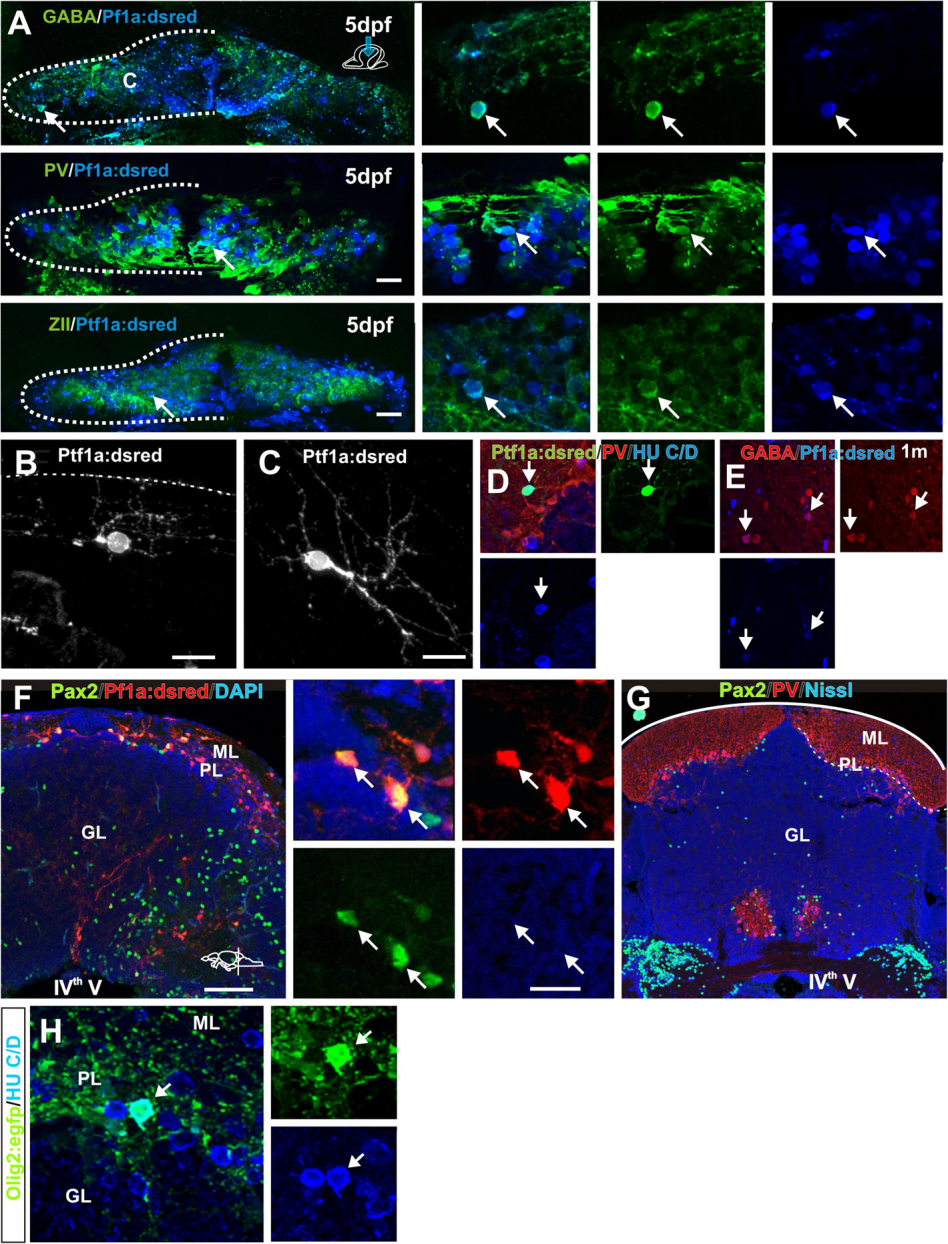
**Cerebellar progenitors gives rise to distinct cell lineages.** (**A**) Co-localization (arrow) of *Ptf1a:DsRed* (blue) and GABA, PV or ZII (green) in inhibitory neurons in the cerebellum of a 5-day-old larvae; (**B,C**) *Ptf1a:DsRed*+ cells showing morphologies of differentiating stellate and Golgi neurons; (**D**) A PV- (red) *Ptf1a:DsRed*+ (green) and HU C/D+ (blue) stellate cell in the ML; (**E**) Co-localization (arrows) of *Ptf1a:DsRed* (blue) and GABA (red) cells in the cerebellum of a 1-month-old juvenile zebrafish; (**F**) Cross section of the juvenile cerebellum showing abundant co-localization (white arrow) of *Ptf1a:DsRed* (red) and Pax2 (green); (**G**) Overview of a cross section of the adult cerebellum showing Pax2 (green) labeled Golgi neurons in the GL and PL. Purkinje neurons in the PL are labeled with parvalbumin (red); (**H**) Co-localization (arrow) of *Olig2:egfp* (green) and HU C/D (blue) cell in the PL of an adult zebrafish.

**Figure 6:**
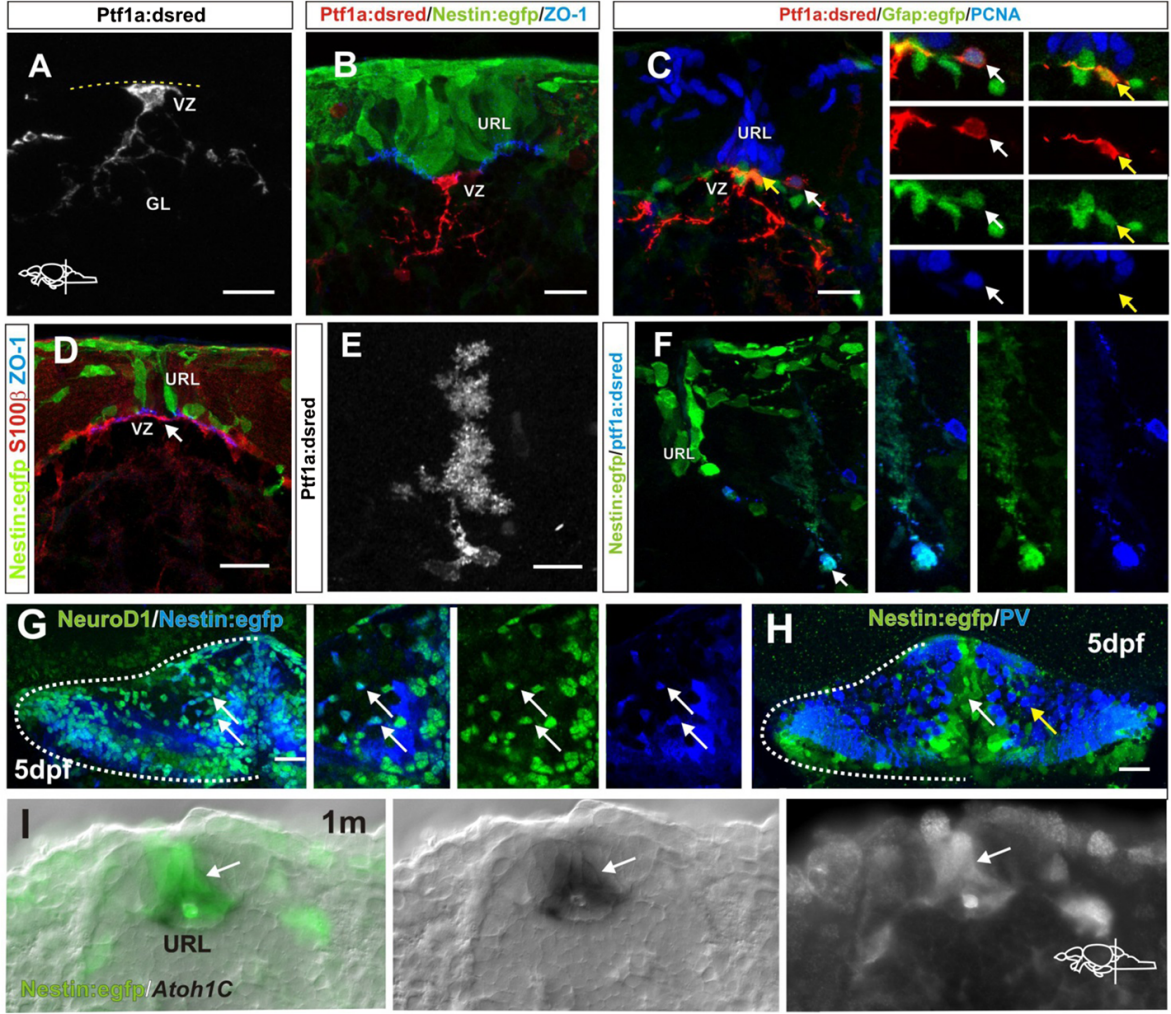
**Morphology of *Ptf1a:DsRed *cells in the VZ.** (**A**) High magnification of a ventricularly located *Ptf1a:DsRed*+ cell with radial morphology in a juvenile zebrafish; (**B**) A *Ptf1a:DsRed*+ cell (red) with a radial morphology located at the ventricle, ventral to the *Nestin:egfp*+ cells (green) in the URL. Ventricle and apical junctions are outlined with ZO-1 (blue); (**C**) *Ptf1a:DsRed*+ and *gfap:egfp+* cells (green) at the ventricle of the VZ. Proliferating cells in the progenitor niche are labeled with PCNA (blue) and glia are labeled by *gfap:egfp* (green). The white arrow depicts a proliferating *Ptf1a:DsRed*+and PCNA+ cell located lateral to the PCNA+ cells in the URL. The yellow arrow depict a horizontally oriented *Ptf1a:DsRed*+ and *gfap:egfp+* cell at the ventricle; (**D**) Flat horizontally oriented s100β+ cells (red) are lining the VZ of the progenitor niche in adult zebrafish. The ventricle is outlined with the junctional marker ZO-1 (blue); (**E**) A *Ptf1a:DsRed*+ Bergmann glia in the adult cerebellum; (**F**) A *Ptf1a:DsRed*+ and *Nestin:egfp*+ Bergmann glia lateral to the progenitor niche (arrow); (**G**) Co-localization (arrows) of *Nestin:egfp* (blue) and NeuroD1 (green) in the cerebellum of a 5 day old larvae; (**H**) No overlap between *Nestin:egfp* (green, white arrow) cells and PV+ cells (blue, yellow arrow) is seen in the cerebellum of a 5-day-old larvae; (**I**) Overlapping expression of *Atoh1c* and *Nestin:egfp* in the URL. C: Cerebellum, GL: Granule cell layer, URL: Upper rhombic lip, VZ: Ventricular zone.

The PCNA-, *Ptf1a:DsRed*+ and *Olig2:egfp*+ cells in the parenchyma displayed morphologies of differentiating neurons and glia (Figure 5). Next we examined if PCNA- and *Nestin:egfp+*, *Ptf1a:DsRed*+ or *Olig2:egfp*+ cells co-localized with glial and neuronal markers. Egfp protein persists in zebrafish tissue for at least 24 hours while DsRed persists for days, therefore, they can be used to transiently follow cell fates. γ-aminobutyric acid (GABA) was used to identify all inhibitory neurons, while parvalbumin (PV) and ZebrinII (ZII) were used to label Purkinje cells. In juvenile zebrafish the *Ptf1a:DsRed*+ cells co-localized with GABA, PV and ZII (Figure 5A). In general, very few *Ptf1a:DsRed*+ cells were found in the parenchyma of young and adult fish and the majority of the PCNA-/*Ptf1a:DsRed*+ cells displayed morphological characteristics and marker expression typical of differentiating neurons and glia (Figure 5B,C, Figure 6E). In young and adult zebrafish of the *Ptf1a:DsRed*+ cells co-localized with the neuronal markers HU C/D and GABA (Figure 5D,E). In the adult, we did not detect any *Ptf1a:DsRed*+ cells with large somas and dendrites characteristic for Purkinje cells (Figure 5B,C). In rodents the progenitors for cerebellar inhibitory interneurons can be distinguished by their expression of Pax2 and in the mature cerebellum Pax2 is expressed by Golgi neurons [35,36]. In agreement with this, we detected extensive co-labeling of the *Ptf1a:DsRed*+ cells with Pax2 in the juvenile zebrafish (Figure 5F). In the adult cerebellum Pax2+/PV- putative Golgi cells were detected in the PL and GL (Figure 5G). The majority of *Olig2:egfp*+ cells in juveniles and adult were in the PL and co-localized with the pan-neuronal marker HU C/D (*elavl3*, Figure 5A). This is in agreement with previous studies that have identified these cells as eurydendroid cells [23,34].

The morphology of *Ptf1a:DsRed*+ cells at the ventricle changed in morphology from radial in juvenile fish to multipolar and flat epithelial morphology in young and adult zebrafish (Figure 6A-D). The morphological change correlated with the proliferative quiescence and the expression of ependymal and glial markers GFAP and s100β (Figure 6C,D). In addition, *Ptf1a:DsRed*+ and *Nestin:egfp+* Bergmann glia was detected in the cerebellar parenchyma and laterally in the progenitor niche (Figure 6E,F). Interestingly, rare proliferating *Ptf1a:DsRed*+ cells were also seen lateral to the progenitor niche (Figure 6C).

We have previously shown that *Nestin:egfp*+ cells in the adult cerebellum express granule cell markers such as NeuroD1, Pax6+, *reelin* and *Zic1,3*[19]. In agreement with this, *Nestin:egfp+* cells in the juvenile cerebellum expressed NeuroD1 but did not co-localize with PV+ cells, suggesting that *Nestin:egfp* primarily labels granule cell progenitors (Figure 6G-H). The transcription factor Atonal1 is required for the genesis of granule cells [37]. Given that *Nestin:egfp* labels granule cell progenitors in the cerebellum it was puzzling that we previously did not detect expression of the zebrafish Atonal1 paralogs *Atoh1a* and *Atoh1b* in the *Nestin:egfp* granule progenitors of adult fish [19]. Recently a third *Atoh1* paralog, *Atoh1c*, was identified and found to be expressed in the zebrafish cerebellum [25,31]. *In situ* hybridization of *Atoh1c* in the *Nestin:egfp* transgenic line showed that *Atoh1c* was expressed in *Nestin:egfp+* cells within the progenitor niche of the juvenile cerebellum (Figure 6I). The *Atoh1c* expression in the adult zebrafish cerebellum was very low.

### Not all cerebellar cell types are homeostatically produced throughout life in zebrafish

**Figure 7:**
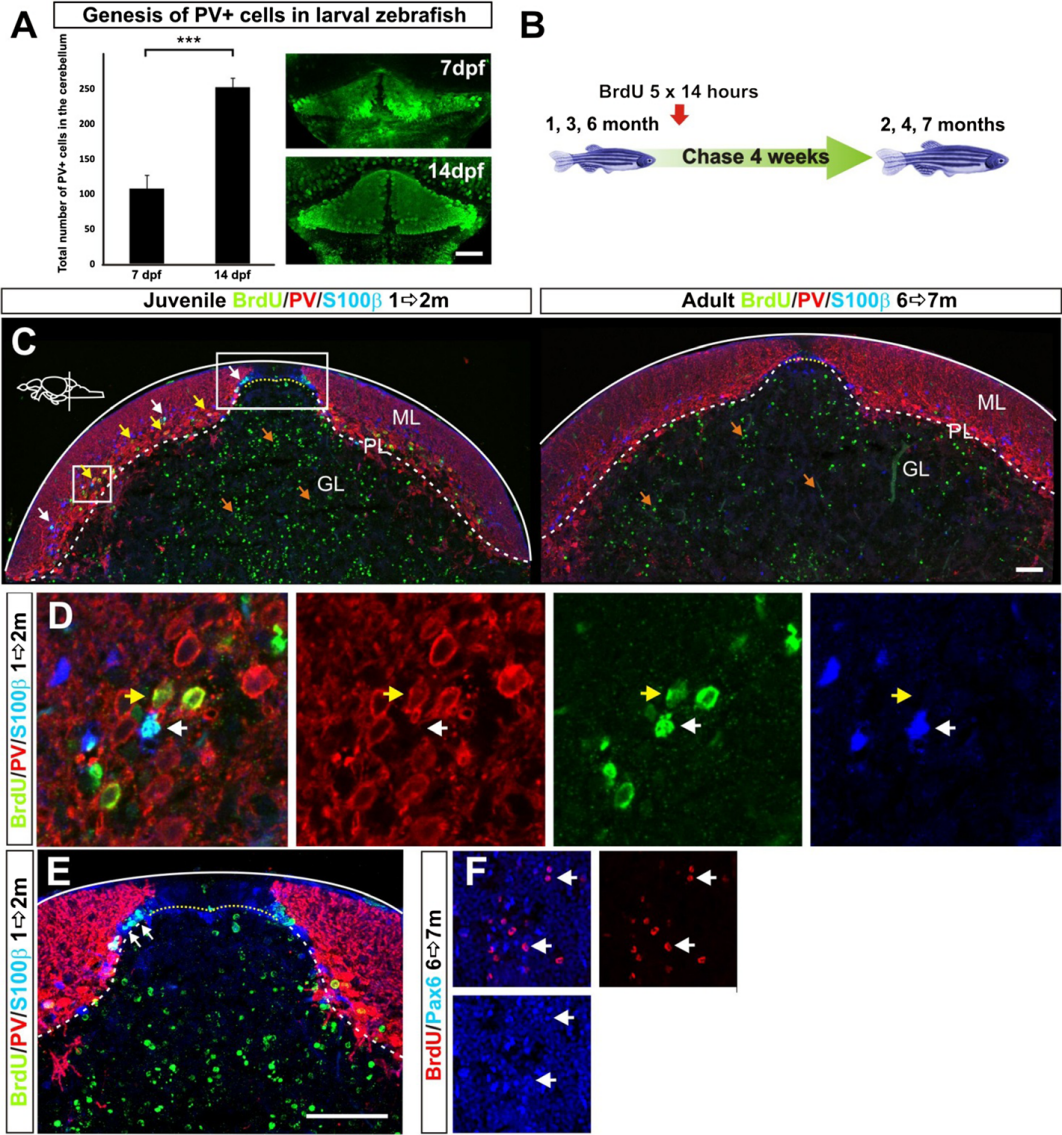
**Generation of cerebellar cell types over time.** (**A**) Quantification of parvalbumin immunopositive cells in the cerebellum of 7- and 14-day-old juvenile zebrafish shows a significant increase of PV+ cells in the cerebellum between 7- and 14-day-old zebrafish (*P* = 0.0003, n = 4). The pictures show a top view of the cerebellum and PV + cells; (**B**) An overview of the BrdU pulse chase experiments. To maximize the labeling of dividing cells the zebrafish were given five consecutive 14 hour pulses of BrdU. The fish were euthanized four weeks after the last BrdU pulse; (**C**) Confocal maximum projections of juvenile and adult cerebellar cross sections showing a notable production of cells in the juvenile and adult zebrafish cerebellum. PV+ Purkinje cells (red) and their processes are seen in in the PL. Scattered s100β+ glia (blue) are seen in the PL and ML. In the juvenile fish high numbers of BrdU+ cells (green) are found both in the GL (orange arrows) and PL (white and yellow arrows). In the adult cerebellum BrdU+ cells are confined to the GL (orange arrows); (**D**) High magnification of a single confocal plane of the boxed area in **C** showing a s100β+ glia co-localizing with BrdU (white arrow) and a PV+ Purkinje neuron co-localizing with BrdU (yellow arrow) in a juvenile zebrafish; (**E**) High magnification maximum projection of the boxed area in **C** showing s100β+ Bergmann glia lateral to the URL progenitor niche (yellow hatched line) co-localizing with BrdU (white arrows); (**F**) High magnification of BrdU+ and Pax6+ granule cells (white arrows) in the GL.

**Figure 8:**
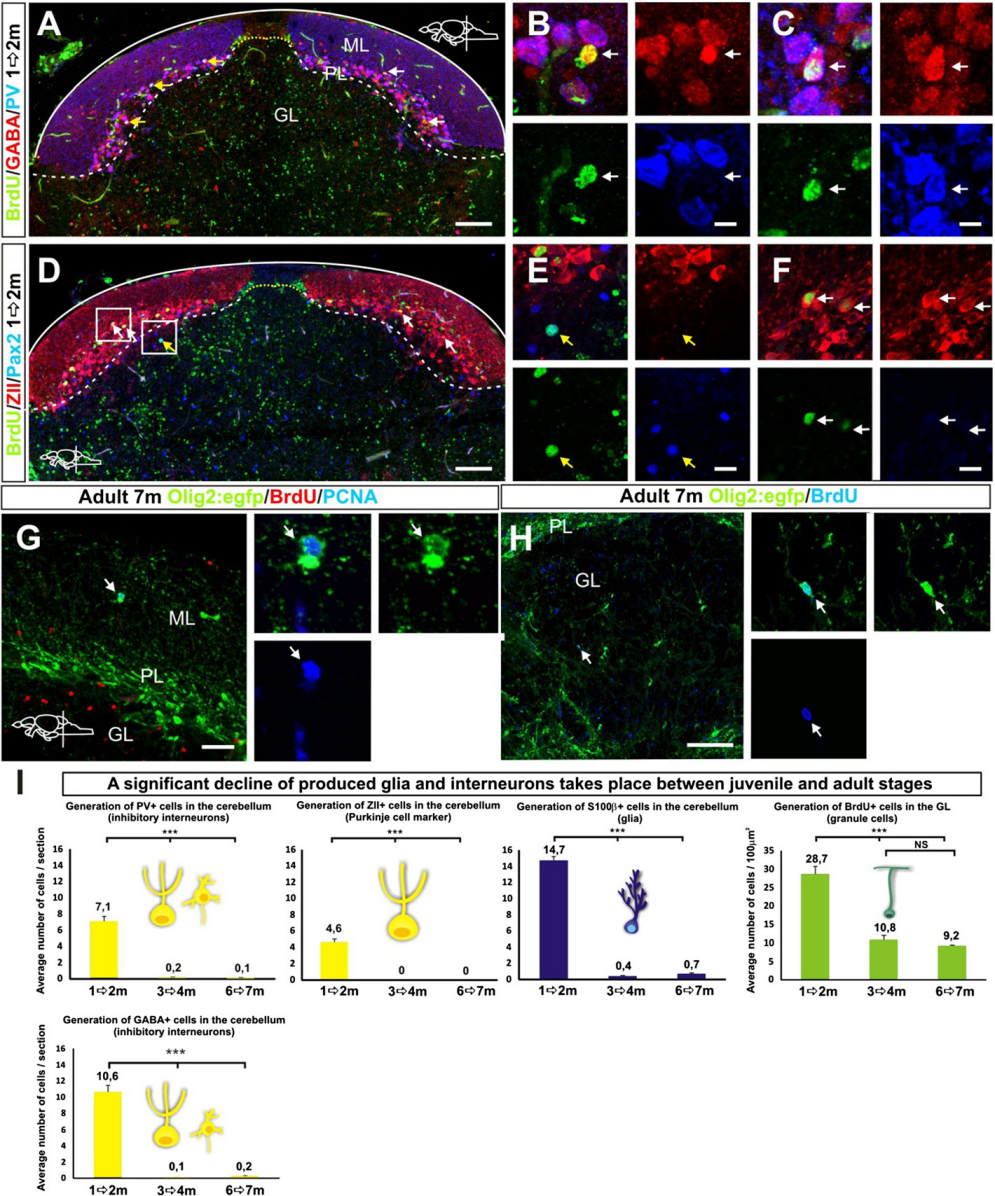
**Generation of different cerebellar cell types.** (**A**) Confocal maximum projection of a cerebellar cross section in a juvenile zebrafish showing BrdU labeled GABA+ inhibitory neurons (white arrows) and BrdU+/GABA+/PV+ neurons (yellow arrows); (**B**) High magnification of a single confocal plane showing a neuron co-localizing with BrdU and GABA (white arrow); (**C**) High magnification of a single confocal plane showing a neuron co-localizing with BrdU, GABA and PV (white arrow); (**D**) Confocal maximum projection of a cerebellar cross section showing BrdU labeled ZII+ Purkinje neurons (white arrows) and a Pax2+ Golgi neuron (yellow arrow); (**E**) High magnification of a single confocal plane showing a Golgi neuron co-localizing with BrdU and Pax2 (white arrow); (**F**) Magnified single confocal plane showing Purkinje neurons co-localizing with BrdU and ZII (white arrows); (**G**) A rare proliferating oligodendrocyte progenitor (PCNA/*Olig2:egfp*+) detected in the cerebellar parenchyma (arrow); (**H**) A putative oligodendrocyte labeled with BrdU+and *Olig2:egfp*+ detected in the brain parenchyma six weeks after the BrdU pulse; (**I**) Quantifications of the cell types produced in the cerebellum of juvenile and adult zebrafish after four weeks BrdU pulse chasing. A significant decline of cerebellar inhibitory neuron and glia production between juvenile and adult stages is detected while granule cell production declines during juvenile stages but is still maintained at a high level in the adult zebrafish (n = 5, *P* <0.001).

Our analysis of proliferative active progenitor types and marker expression suggested that mainly granule cells, inter-neurons and Bergmann glia are produced in juvenile and adult fish. To examine what cerebellar cell types are produced at different ages, we carried out BrdU pulse chase experiments and quantifications using cellular markers. During early cerebellar development in chick and rodents, Purkinje cells are produced during a relatively short time window. We first quantified the total number of the parvalbumin+ Purkinje cells in the whole cerebellum of 7- and 14-day-old larval zebrafish. The total number of parvalbumin+ cells in the cerebellum doubled between 7 and 14 days post-fertilization, showing that Purkinje cells were produced during larval stages (Figure 7A). This suggested that diverse cerebellar cell types were produced in juvenile zebrafish. Next, we performed a series of BrdU pulse-chase experiments in zebrafish of different ages (1, 3 and 6 months, Figure 7B). The zebrafish were given extensive consecutive pulses of BrdU (5 × 14 hours) by immersion. After four weeks of chase time, we performed a quantitative co-localization study with BrdU and cellular markers to identify which cell types had been produced. GABA was used to identify all inhibitory neurons, while parvalbumin, ZII and Pax2 were used to label Purkinje and Golgi neurons, respectively. S100β labeling identified Bergmann glia, and granule cells were identified based on their location, expression of Pax6, and the lack of glial and inhibitory neuron marker expression. The BrdU pulse chase experiments showed that different inhibitory BrdU+/GABA+ cell types were produced in the juvenile fish. In one month old zebrafish the majority of the BrdU+/GABA+ cells were positive for the Purkinje cell markers PV and ZII (Figure 7C, Figure 8A-F). The BrdU+ cells negative for PV or ZII co-localized with the Golgi cell marker Pax2 (Figure 8E). Quantification of BrdU+/GABA+ and BrdU+/PV+ cells showed a significant decline in the genesis of inhibitory neurons in the 3- and 6-month-old fish (*P* <0.001, Figure 1). Altogether, only a low number of inhibitory neurons and glia were produced in the adult zebrafish brain (<10 cells of respective cell type/brain, n = 5). Interestingly, no BrdU+ and PV+ or ZII+ Purkinje cells were detected in the adult cerebellum, suggesting that no or very few Purkinje cells were produced in the sexually mature zebrafish (Figure 8I). Eurydendroid cells in the PL were identified by using the *Olig2:egfp* transgenic line that labels eurydendroid cells and oligodendrocytes in the adult zebrafish cerebellum [23,34]. We detected a few proliferating PCNA+/*Olig2:egfp+* cells in the cerebellar parenchyma (Figure 8G). Further, we detected very few BrdU+/*Olig2:egfp+* cells in the parenchyma six weeks after the last BrdU pulse (Figure 8H). We did not identify any BrdU+/HU C-D+/*Olig2:egfp+* cells showing that very few or no eurydendroid cells were produced in adult zebrafish (n = 5). A considerable number of granule cells were produced in both the juvenile and the adult zebrafish cerebellum (Figure 7C,F). However, there was a significant decline in the number of granule cells produced during the transition from juvenile to adult (Figure 8I). BrdU+/S100β+ Bergmann glia were detected after BrdU chasing in the PL and ML. In particular, BrdU+/S100β+ cells were localized in a row of cells lateral to the progenitor niche (Figure 7E). The production of Bergmann glia lateral to the progenitor niche was supported by the observation of proliferating and differentiating *Nestin:egfp+/Ptf1a:DsRed+* cells in this location (Figure 6C,F). Quantification of BrdU+/S100β+ cells showed that there was a significant decline in the genesis of Bergmann glia in the adult brain (*P* <0.001, Figure 8I).

## Discussion

**Figure 9:**
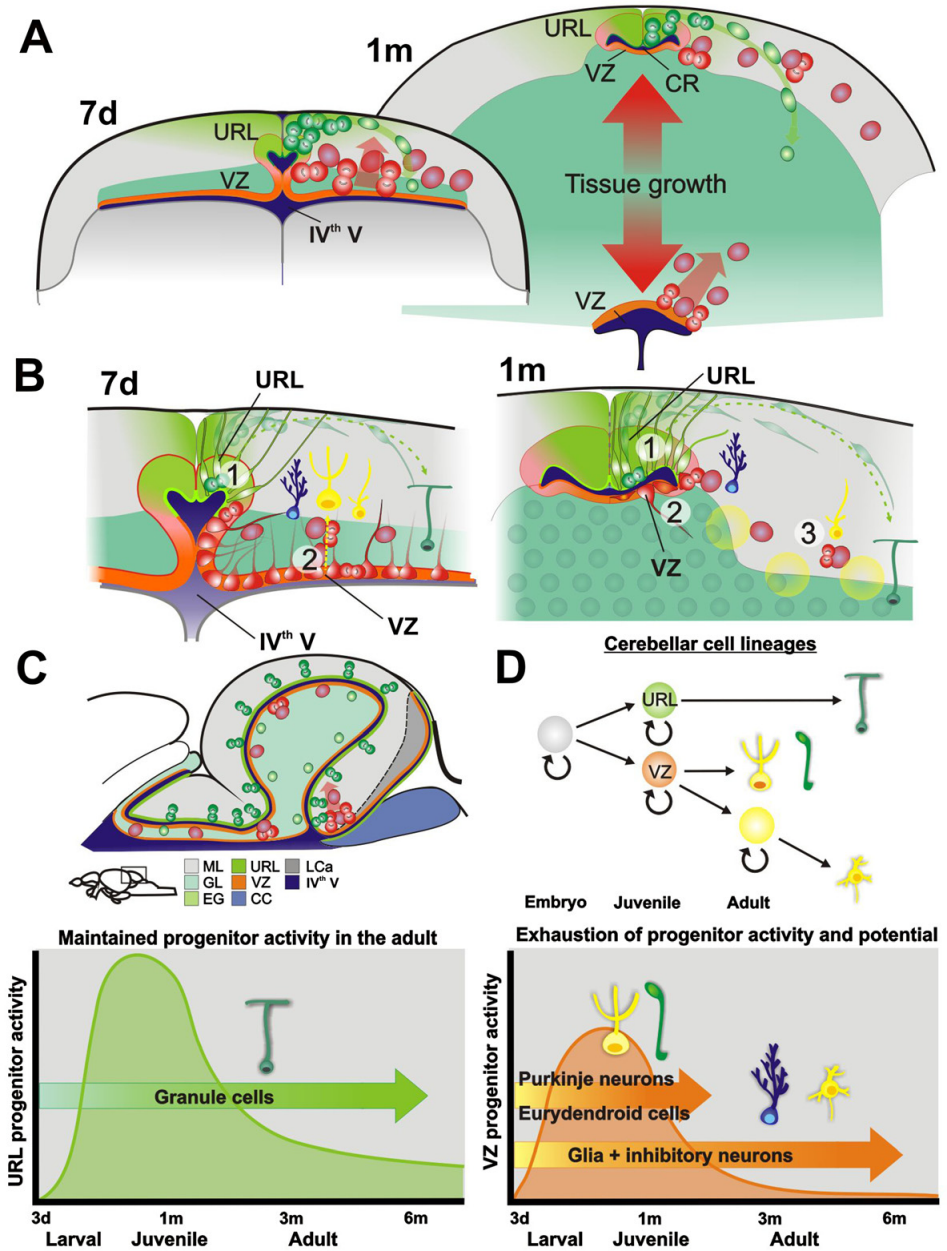
**Summary of the cerebellar progenitors and the progenitor niche in zebrafish.** (**A**) During larval and juvenile stages the URL and a portion of the VZ together with a part of IV^th^ ventricle (cerebellar recessus, CR) is displaced from the rest of the ventricle through tissue growth (red opposing arrows). The neural progenitors in the URL (dorsal part) remain active throughout life, while the VZ progenitors (ventral part) largely become quiescent in the adult. During larval and juvenile stages progenitors delaminate from the VZ into parenchyma. The highest density of proliferating VZ progenitors is found at the interface of the caudal lobe (best seen in **C**); (**B**) Schematic view of the cellular arrangement of the cerebellar progenitor niche. **1.** Granule cell stem and progenitor cells (green) are found dorsal to the cerebellar recessus in the URL. The granule precursors migrate to the GL and differentiate into granule neurons. **2.** Ventricular zone derived progenitors (orange) are found ventral to the recessus. Proliferating and differentiating VZ progenitors migrate to the cerebellar parenchyma during larval and juvenile stages. Most of the cells in VZ of the mature cerebellum express glial and ependymal markers and display epithelial-like morphology. Bergmann glia and inhibitory inter-neurons are produced at the interface between the URL and VZ. **3.** Very rare progenitors reside in the cerebellar parenchyma. (**C**) Schematic parasagittal view of the cerebellum and the distribution of progenitors; (**D**) The URL derived progenitors remain active into adulthood, while the proliferative activity of VZ derived progenitors virtually is exhausted during juvenile stages. Purkinje neurons and Eurydendroid cells are produced up to 1 month. In sexually mature zebrafish only few inhibitory neurons and glia are produced. Production of granule neurons is maintained throughout life in zebrafish.

In contrast to mammals, teleost fish display widespread post-embryonic neurogenesis and net brain growth throughout life. We wanted to address if diverse neural stem and progenitor types remain active throughout life in teleost fish and if all major cell types are generated. We followed how the cerebellar stem and progenitor cell populations progress from the embryo to adult. In agreement with previous studies, we show that distinct neural stem and progenitor cell populations that give rise to specific cell lineages are formed during early development in the VZ and URL of the zebrafish cerebellum [22,25,29]. In the juvenile and adult zebrafish, the cells of the VZ and URL form a distinct structure, the cerebellar progenitor niche, around the IV^th^ ventricle (Figure 9). We previously showed that progenitors in the URL and the dorsomedial part of the IV^th^ ventricle, the cerebellar recessus, are separated from the rest of the ventricle through morphogenetic movements and tissue growth [19]. The stem and progenitor cells derived from the URL are maintained dorsal to the recessus, while VZ derived cells and progenitors are found ventral. By following and quantifying the proliferative activity of the VZ and URL progenitor populations we discovered that the proliferative activity and progenitor marker expression drastically declined in juvenile zebrafish before they reached sexual maturity. This correlated with the diminished repertoire of produced cell types. Our data suggest that neural progenitors derived from the VZ turn into quiescent epithelial-like glia or alternatively are exhausted (undergo differentiation) during juvenile stages. The loss of proliferative activity of the VZ progenitors during juvenile stages was directly linked to the diminished production and repertoire of specific cell lineages in the adult cerebellum. We detected only low production of Golgi, stellate cells, Bergman glia and oligodendrocytes after extensive BrdU pulse chase experiments in mature fish (<20 cells/brain of respective cell type). Importantly, we did not find any new Purkinje or eurydendroid cells in BrdU pulse chased fish older than 3 months. In rodents, the VZ derived progenitors of cerebellar inhibitory interneurons can be distinguished by their expression of Pax2 [35,36]. In agreement with this, many of the *Ptf1a:DsRed*+ cells in the cerebellar parenchyma co-localized with Pax2 in juvenile fish, suggesting that many of the *Ptf1a:DsRed*+ at this time point already were specified as progenitors of inhibitory interneurons. Thus, the shift from the production of Purkinje cells to Golgi and stellate cells that took place in juvenile zebrafish was accompanied by the expression of Pax2. Further, Bergman glia cells were preferentially produced lateral to the progenitor niche at the interface between the URL and VZ (Figure 7E). In contrast to the VZ progenitors, the URL derived stem and progenitor cells remained proliferatively active in the dorsal cerebellum and a copious production of granule cells was detected in adult and aging zebrafish. However, we detected a very noticeable decline in the proliferative activity of the URL progenitor and granule cells between the juvenile and adult zebrafish. During mammalian development, different cerebellar cell types are produced in a strict temporal order from increasingly committed progenitors. Purkinje neurons and deep cerebellar nuclei neurons (eurydendroid cell equivalents in teleost) are produced early during development, while inhibitory inter-neurons, granule cells and glia are produced late [27]. We could detect a similar temporal order in zebrafish: Purkinje cells and eurydendroid cells were only produced during larval and juvenile stages while inhibitory inter-neurons, granule cells and glia still were produced in the adult zebrafish. It is important to note that the relative ratio between different cell types, such as between granule and Purkinje cells, changes during the life time of zebrafish. The granule cells give rise to parallel fibers that ascend through the molecular layer where they synapse to the dendritic branches of Purkinje cells and stellate cells [38]. The changing ratio could have profound effects on the function of the cerebellar circuitry. We hypothesize that the limited number of Purkinje cells may accommodate additional neuronal input by increasing the available surface area for interaction by increased dendritic branching or by a general increase in cell volume. Alternatively, the dendritic space may initially not be fully occupied and the occupancy is gradually filled when new granule cells are added. Taken together, our results suggest that neural progenitor types retain their specificity for distinct neuronal subtypes throughout life in fish. We propose that only specific subtypes of brain cells are continuously produced in the fish brain. This further suggests that the production of specific cell types could be differentially regulated during the life time. In a wider sense this implies that the post-embryonic neurogenesis in fish is linked to the production of particular neurons involved in specific brain functions, rather than a general, indeterminate growth of the CNS and all of its cell types. What the functional implications of the newly produced cells are and how they are able to integrate and reinforce specific neural circuits of the mature fish brain remains to be studied.

During development of amniotes granule cell precursors migrate from the URL to the cerebellar surface where they transiently form a highly proliferative second germinal zone, the external granule layer. A similar structure is apparently lacking in zebrafish [19,31]. This study elaborates on this by examining the cerebellar progenitor populations in a wide range of larval and juvenile stages. We did not detect a developmental or juvenile phase where cells would pile up and form a transient amplifying structure like the external granular layer in birds and mammals. Sonic hedgehog acts as the major mitogenic signal for granule precursors in the external granule layer in birds and mammals [39,40]. However, sonic hedgehog signaling is absent in the developing zebrafish and dogfish (shark) cerebellum [31]. Atoh1 expression is hallmark of the external granule cell layer in chick and mouse, and Atoh1 is crucial for the development of granule cells. In zebrafish three Atoh1 paralogs have been identified [25,31]. Interestingly, the Atoh1 paralogs are differently expressed during cerebellar development [25,31]. Atoh1a and Atoh1b are expressed up to larval stages, while Atoh1c starts to be expressed during larval stages and is highly expressed in the juvenile zebrafish and lowly in the adult cerebellum. However, none of them are expressed in a structure reminiscent of an external granule cell layer. Taken together, the morphological and molecular data collectively suggest that the advent of a secondary zone of transient amplification (*e.g.*, the external granule cell layer) seems to be an amniote-specific developmental adaptation because shark, teleost fish and frogs lack an obvious external granule cell layer [19,31,41].

The lack of an obvious amplification layer further suggests that the production of granule cells in zebrafish is controlled on the level of the primary progenitors [19]. Not much is known about signals that regulate proliferation and differentiation of the primary progenitors in the cerebellum. Fgf signaling is crucial during cerebellar development. Fgf signaling components are expressed in the cerebellum and Fgf signaling is required for the maintenance and proliferation of the adult cerebellar progenitors [19]. In the future it will be important to identify mechanisms that coordinate the precise cellular output in the zebrafish cerebellum. Although a transient amplifying external granule layer as such does not exist in the zebrafish many of the cellular and molecular attributes are evolutionarily conserved [19,24,31]. The cerebellar granule precursor in developing and adult zebrafish express markers observed in external granule layer of rodents and chick, such as Atoh1a-c, Zic1, Zic3, Pax6 and NeuroD [24]. The granule cell migration pattern is well conserved between zebrafish and rodents [19,27]. Initially granule precursors migrate dorsally to the cerebellar surface from the germinative zone. During this stage massive secondary amplification takes place and the external granule layer is formed in rodents. In zebrafish dividing cells can be detected but the amplification is minor and no clearly discernible amplifying layer is formed. This is followed by tangential and ventrolateral movements and a final inwards migration into the granule cell layer. Overall, the cerebellar development program is highly conserved in vertebrates in terms of morphogenesis, cell migration, ontogeny of cell types and expression of molecular markers.

Our results show that neural stem cells in zebrafish are regionally specified during development as in rodents [1]. Furthermore, our results also support and favor the newly proposed hypothesis of disposable neural stem cells, *i.e.*, that stem and progenitor populations are gradually exhausted [5] over the hypothesis suggesting that stem cells are indefinitely maintained [43]. Overall, our data supports the view that adult neural stem and progenitor cells in general are diverse and restricted in potential over the view of uniform stem cells with a broad potential. The ability of zebrafish to regenerate tissues and organs such as the retina, fin and heart is thought to depend on cellular de-differentiation and this has led to the idea that de-differentiation is a crucial mechanism of regeneration zebrafish [44]. However, we recently found that radial glia act as the major progenitor population during neuronal regeneration in the adult telencephalon of zebrafish [5]. Therefore, an important question for a future study is whether the stem and progenitor cells that persist in the cerebellum retain the capacity to produce all the cell types after injury or whether other cellular mechanisms take place. In general, the widespread adult neurogenesis along the brain axis in zebrafish provides exciting potential as a new powerful model for studying brain regeneration and neuronal stem cell diversity. Understanding the molecular basis of *in vivo* neural stem cell heterogeneity and plasticity in vertebrates may be of great relevance for future therapeutical approaches. How new neurons integrate and reinforce specific neural circuits in the zebrafish brain and how this relates to the modulation of different behaviors will also be an interesting avenue for future studies.

## Conclusions

Our results in the zebrafish cerebellum show that neural stem and progenitor cell types are specified early during development and produce distinct cell lineages and sub-types of brain cells. Therefore we propose that only specific subtypes of brain cells are continuously produced throughout life in the teleost fish brain. This implies that the post-embryonic neurogenesis in fish is linked to production of particular neurons involved in specific brain functions, rather than a general, indeterminate growth of the CNS and all its cell types. Neural stem and progenitor cells in zebrafish are regionally specified during development as in rodents. Further, our data support the proposed hypothesis of disposable neural stem cells, *i.e.*, that stem and progenitor populations are gradually exhausted. Overall, our data supports the view that adult neural stem and progenitor cells in vertebrates may in general be much more diverse and restricted in potential than previously anticipated.

## Methods

### Zebrafish maintenance

Zebrafish were bred and maintained according to standard procedures [45]. All animal procedures were approved by the Regierungspräsidium Dresden (permit AZ 24D-9168.11-1/2008-1 and −4). Wild type experimental fish were from the *gol-b1* line in the AB genetic background. The fish were raised at a density of 50 to 60 fish per tank. The larvae and juveniles were kept in 7 L mouse cages and moved to 11 L glass tanks at 1 month of age. Fish of either sex were used. For the quantifications and lesion experiments: 1-month-old juvenile fish had a body length of 9 mm (± 0.5 mm), 3-month-old young adults of 19 mm (± 1 mm) and 6-month-old adult fish of 30 mm (±2 mm).

### Transgenic lines

To generate the *Tg(−5.5Ptf1a:DsRed)*^*ia6*^ transgenic line a 5.5 kb fragment of the promoter region of Ptf1a was cloned upstream to DsRed2 in pT2AL200R150G vector [46]; 25 to 100 pg of linearized vector DNA and 50 pg of Tol2 transposase mRNA were injected into fertilized eggs at one cell stage. F0 were raised and incrossed. F1 progeny were identified by visual screening for DsRed expression. The *Tg(Nestin:egfp)* zebrafish line expresses EGFP under the control of 41 kb of the nestin locus and it is recapitulating the endogenous expression of nestin [47].

### BrdU labelling

To label cycling cells, zebrafish were immersed in 7.5 mM BrdU (Sigma) solution [10]. BrdU was dissolved in E3 medium and the pH adjusted to 7.5.

### Tissue preparation

Exposed brains in skull were fixed at 4°C overnight in 2 to 4% paraformaldehyde/0.1 M PB (pH 7.5). They were washed twice with PB and transferred for decalcification and cryoprotection to 20% sucrose/20% EDTA in 0.1M PB (pH7.5). Brains were frozen in 7.5% gelatin/20% sucrose and cut at 16 μm. Sections were stored at −20°C.

### Immunohistochemistry

Immunohistochemistry was carried out as previously described [48]. Briefly, primary and secondary antibodies were incubated in PBS with 0.3% TX. Primary antibodies were incubated overnight at 4°C and secondary antibodies for 1 h RT. The slides were then washed in PBS TX and mounted. We used primary antibodies to parvalbumin (mouse, Chemicon, Mab1572, 1/5000), neuroD1 (mouse, Abcam, ab60704, 1/2000), Pax6 (rabbit, Babco, PRB-278P, 1/750), HU-C/D/Elavl3 (mouse, Invitrogen, A-21271, 1/250), PCNA (mouse, clone PC 10, Dako cyto, 1/1000), S100β (rabbit, Dako cyto, Z 0311, 1/2000), GABA (rabbit, Sigma, A-2052, 1/10000), Zebrin II/Aldolase C (mouse, kindly provided by Richard Hawkes, 1/250), Pax2 (rabbit, Covance, PRB-276P, 1/750), GFP (chick, mouse, Invitrogen, A10262, 1/1000), DsRed (rabbit, Clontech, 632496, 1/1000), BrdU (rat clone BU1/75 (ICR1), Serotec, MCA2060, 1/500). For detection Alexa 488, 555 and 633 conjugated secondary antibodies were used (Invitrogen, 1/750).

### *In vivo* imaging, image acquisition and processing

Live embryos or zebrafish were anesthetized with 0.1% Tricaine (Sigma), mounted in 1.5% LMP agarose in embryo medium 3 (E3) and imaged with a Leica TCS SP5 confocal microscope using HCX APO L 20/0.5 NA, HCX APO L 40/0,8 NA, HCX APO L 63/0.9 NA dipping objectives. Other images were taken with a Leica TCS-SP5 confocal microscope using HC PL APO CS 20x/0.7 NA, HCX PL APO 40/1.25 NA and HCX PL APO 63x/1.2 NA objectives. To minimize cross-talk between the channels in multi colored specimens sequential image acquisition was performed. The images were processed using ImageJ v.1.44 (http://rsb.info.nih.gov/ij/) and Adobe Photoshop CS4. Figures were assembled using Adobe Photoshop CS4 and Corel Draw X3.

### Quantification and statistical analysis

We quantified the number of BrdU+ cells in every fourth section (16 μm) throughout the whole length of the cerebellar corpus. For the co-localization studies with cellular markers, co-localization was verified by analyzing high resolution confocal stacks. The optical sections were taken with 0.5 to 1 μm intervals using 40× (1.25 NA) or 63× (1.2 NA) objectives. Data are presented as mean ± SD. For all analyses a normal distribution of values was assumed. Comparison between the groups was made by unpaired two-tailed Student’s *t*-test or one-way ANOVA analysis with Tukey's Multiple Comparison post-test. A *P* value <0.05 was considered to be statistically significant. Statistical analysis was performed with GraphPad Prism 4.03.

## Abbreviations

IVth V: IV^th^ ventricle; CR: Cerebellar recessus; GL: Granule cell layer; ML: Molecular layer; PL: Purkinje cell layer; URL: Upper rhombic lip; VZ: Ventricular zone.

## Competing interests

The authors declare no financial competing interests or other conflict of interest.

## Authors’ contributions

JK designed and performed the experiments and wrote the manuscript. VK co-performed experiments and co-wrote the paper. MB directed the research, co-designed the experiments, co-wrote the paper and secured the funding for this research. AF and FB designed and made the Tg(−5.5Ptf1a:DsRed) transgenic line and commented on the manuscript. All authors read and approved the final manuscript.
